# CpG oligodeoxynucleotides enhance chemosensitivity of 5-fluorouracil in HepG2 human hepatoma cells via downregulation of the antiapoptotic factors survivin and livin

**DOI:** 10.1186/1475-2867-13-106

**Published:** 2013-10-26

**Authors:** Sheng-ran Liang, Guang-rui Hu, Li-juan Fang, Su-jing Huang, Jin-song Li, Ming-yi Zhao, Min-jie Meng

**Affiliations:** 1 School of Basic Courses, Guangdong Pharmaceutical University, Guangzhou 510006, P. R. China; 2 School of Traditional Chinese Medical, Guangdong Pharmaceutical University, Guangzhou 510006, P. R. China; 3 School of Life Science and Biopharmaceutical, Guangdong Pharmaceutical University, Guangzhou 510006, P. R. China

**Keywords:** CpG-ODN, Human hepatoma cells, Chemosensitizer, Apoptosis, Cell cycle arrest, Livin, Survivin

## Abstract

**Background:**

Recent studies indicated that a synthetic oligonucleotide containing un-methylated CpG oligodeoxynucleotides (CpG-ODN) has a potential function for cancer therapy. In this study, we evaluated the chemosensitizing effects of CpG-ODN in 5-fluorouracil (5-FU)-treated HepG2 human hepatoma cells.

**Methods:**

Cell viability assay were utilized to evaluate the direct cytotoxicity of CpG-ODN in the presence or absence of 5-FU in HepG2 cells, and apoptosis as well as cell-cycle was examined by flow cytometry analysis. The mRNA expression of Bcl-2, Livin and Survivin within HepG2 cells treated with CpG-ODN and/or 5-FU were analyzed by Real Time PCR assay in vitro.

**Results:**

CpG-ODN in combination with 5-FU could decrease cell viability, increase apoptosis and further induce HepG2 cells cycle arrest at S phase when compared with CpG-ODN or 5-FU. CpG-ODN or 5-FU could down-regulate the mRNA expression of Bcl-2 within HepG2 cells. The mRNA expression of Livin and Survivin decreased in cells treated with CpG-ODN alone but increased in cells treated with 5-FU alone. However, CpG-ODN in combination with 5-FU could down-regulate the mRNA expression of Livin and Survivin within HepG2 cells.

**Conclusions:**

Our finding demonstrated that CpG-ODN enhanced the chemosentivity of 5-FU in HepG2 human hepatoma cells at least in part by down-regulating the expression of Livin and Survivin, leading to apoptosis and further inducing cell cycle arrest at S phase. Therefore, CpG-ODN may be a potential candidate as chemosensitizer for human hepatocellular carcinoma.

## Background

Hepatocellular carcinoma (HCC) is the main type of liver cancer and the second leading cause of cancer-related deaths worldwide [1]. It is well known that HCC is one of the malignant tumors with poor chemosensitivity to anticancer agents [2]. To date, the combination therapy with chemotherapeutic agents and immunostimulators, such as 5-fluorouracil (5-FU) and IFN, has been found to be effective in enhancing the HCC-inhibitory effect of chemotherapy [3,4]. Sakabe, T., et al. found some genes that are involved in chemosensitizing the effects of 5-FU and IFN-α/5-FU on HCC cells [5]. Previous study demonstrated that hepatocellular carcinoma (HCC) has high mortality partly due to acquiring drug resistance during chemotherapy treatment [6]. Acquired resistance to 5-fluorouracil (5-FU) is a serious therapeutic obstacle in advanced hepatocellular carcinoma (HCC) patients, but chemosensitizer can partly reverse 5-FU resistance in HCC cells [7]. Therefore, there is an urgent need for the development of a chemosensitizer to increase the sensitivity of tumor cells to chemotherapy within normal dosage.

Recently, a rapid accumulating evidence demonstrated that CpG-ODN have been developed to stimulate the innate immune response in various diseases through the pathogen-associated molecular pattern receptor 9 (TLR9) [8], which are mainly expressed on immune cell [9], are also widely expressed on various tumor cells, including human HCC cells [10]. Previous studies have showed that CpG-ODN can mediate anti-tumor effects on cancer cells due to its direct or indirect effects by inducing release of cytokines, and enhancing immune response [11]. Meanwhile, recent studies suggested that CpG-ODN may be considered as a potential chemosensitizer with weak side effects, such CpG-ODN 1826 [12,13] Clinical studies have also documented that CpG-ODN in combination with chemotherapy cannot only increase the treatment benefit of patients ,but also make patients with well tolerated [14,15]. Although a previous study had demonstrated the benefits of CpG-ODN for HCC treatment [16], the direct cytotoxicity of CpG-ODN against HCC cells and the potential mechanism are not clear.

In our study, we investigated the chemosensitivity of CpG-ODN in HepG2 human hepatoma cells, the HepG2 cells were cultured in vitro, and the cell proliferation, apoptosis, cell cycle and the antiapoptotic factors were tested. The possible molecular mechanisms were investigated.

## Results

### CpG-ODN in combination with 5-FU decreases cell viability in HepG2 cells

**Figure 1:**
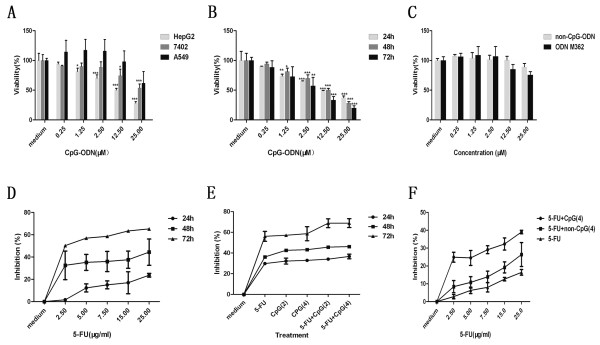
**Effect of CpG-ODN and/or 5-FU treatment on the cell viability in vitro. A**: The cell viability of HepG2 cells, BEL-7402 cells and A549 cells treated with indicated concentration of CpG-ODN for 48 h in MTS assay, cell viability was expressed as the percentage of control cells (medium). **B**: The cell viability of HepG2 cells treated with indicated concentration of CpG-ODN for indicated time in MTS assay, cell viability was then quantified as described above. **C**: The cell viability of HepG2 cells treated with indicated concentration of non-CpG-ODN and ODN M362 for 48 h in MTS assay, cell viability was then quantified as described above. **D**: HepG2 cells were treated with indicated concentration of 5-FU for indicated time in MTS assay, results were presented as the inhibitory ratio of 5-FU treated HepG2 cells. **E**: HepG2 cells treated with indicated concentration of CpG-ODN, 5-FU or CpG-ODN in combination with 5-FU for indicated time, inhibition was then determined by the MTS assay. **F**: The inhibitory ratio of HepG2 cells treated with a rang of 5-FU doses in the presence and absence of 4μM CpG-ODN or non-CpG-ODN for 24h. In this figure, 5-FU, CpG(2) ,CpG(4) and non-CpG(4) represent that the concentration of 5-FU,CpG-ODN , CpG-ODN and non-CpG-ODN is 7.5 μg/ml , 2 μM ,4 μM, 4 μM, respectively. All results shown were means ±SD from three independent experiments. *P<0.05, **P<0.01 and ***P<0.001 versus medium.

Previous studies showed that TLR9 is expressed in human lung cancer A549 cells [17] and human hepatoma HepG2 cells [10]. TLR9 expression and function in BEL-7402 cells, belong to the human hepatoma cell lines, are not reported. The expression of functional active TLR9 in human malignant tumors might affect treatment approaches using CpG-ODN. To evaluate the cytotoxicity of CpG-ODN on HepG2 cells, BEL-7402 cells and A549 cells, cells were incubated with five gradient concentration ranging from 0.25 to 25 μM for 48 h. The viability was determined using CellTiter 96®Aqueous One Solution Cell Proliferation Assay (MTS). The results showed that CpG-ODN significantly decreased the viability of HepG2 cells in a dose-dependent manner. However, CpG-ODN had no effect on the viability of BEL-7402 cells and A549 cells (Figure 1A). These findings indicated that HepG2 cells, but not BEL-7402 cells and A549 cells, are sensitive to CpG-ODN. Taking into account the high inhibition of CpG-ODN on HepG2 cells, and it was selected and used in the subsequent experiments.

Tanaka, J. et al. [10] found that HepG2 cell following incubation with the type C CpG oligonucleotide (ODN M362: 5′-TCGTCGTCGTTCGAACGACGTTGAT-3′) could promote cell proliferation and survival in human hepatocellular carcinomas. Herein, we compared the cytotoxicity of non-CpG-ODN,CpG-ODN and ODN M362 toward HepG2 cells, cells were treated with non-CpG-ODN, CpG-ODN or ODN M362 at five concentration ranging from 0.25 to 25 μM for the indicated time. The result showed that CpG-ODN significantly reduced the viability of HepG2 cells in time and dose-dependent manner (Figure 1B). However, non-CpG-ODN and ODN M362 didn’t have direct cytotoxicity toward HepG2 cells (Figure 1C). The results showed that CpG-ODN, but not ODN M362 and non-CpG-ODN, can directly mediate cytotoxicity toward HepG2 cells.

To evaluate the inhibition effect of 5-FU treatment on HepG2 cells, the proliferation of cells following treated with 5-FU at designated concentration(2.5,5,7.5,15,25 μg/ml) was detected using MTS assay after incubation for another 24 h、48 h and 72 h. The result showed that 5-FU alone inhibited the proliferation of HepG2 cells in time dependent manner (Figure 1D). In order to evaluate the synergistic effect of CpG-ODN and 5-FU on HepG2 cells, cells following treated with CpG-ODN in presence or absence of 5-FU were carried out in MTS assay. As illustrated in Figure 1E, CpG-ODN in combination with 5-FU could decrease cell viability when compared with CpG-ODN or 5-FU alone. To further determine if CpG-ODN can enhance the chemosensitivity of 5-FU-treated HepG2 cells , the cells treated with a rang of doses of 5-FU in the presence and absence of 4 μM CpG-ODN or non-CpG-ODN for 24 h. Figure 1F showed that treatment with a series of doses of 5-FU in the presence of CpG-ODN( 4 μM) increased the inhibition compared with 5-FU and/or non-CpG-ODN treated groups, further supporting the synergistic effect.

### CpG-ODN in combination with 5-FU affects the cell morphology of HepG2 cells

**Figure 2:**
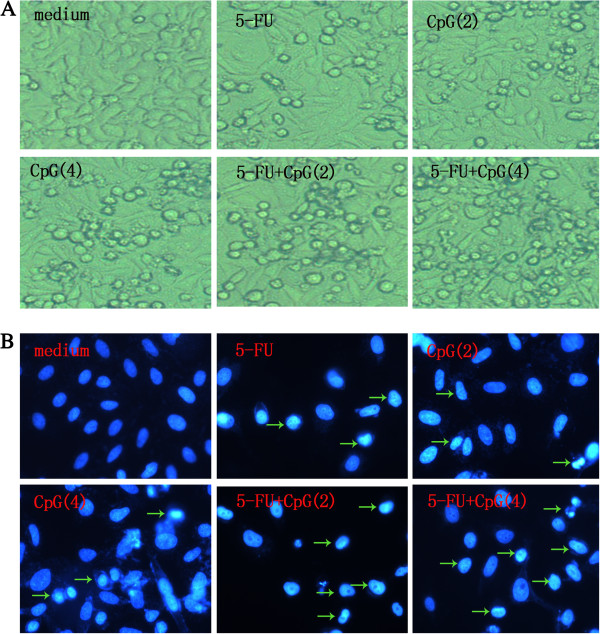
**Effect of CpG-ODN and/or 5-FU treatment on the cell morphology of HepG2 cells. A**: Morphologic changes of HepG2 cells treated with the indicated concentration of CpG-ODN and 5-FU alone or together for 48 h, compared with untreated cells (medium) (100X). **B**: HepG2 cells treated with the indicated concentration of CpG-ODN and 5-FU alone or together for 48 h, then stained with Hoechst 33258(400 X). Condensed and fragmented nuclei in cells were marked by arrow heads. In this figure, 5-FU, CpG(2) and CpG(4) represent that the concentration of 5-FU, CpG-ODN and CpG-ODN is 7.5 μg/ml, 2 μM, 4 μM, respectively.

In order to make sure whether CpG-ODN and/or 5-FU treatment could affect the cell morphology of HepG2 cells, the morphology of cells were observed in the inverted microscope. The microscopic observations revealed that the exposure of HepG2 cells in CpG-ODN in combination with 5-FU for 48h displayed significant morphology alterations. For 7.5μg/ml 5-FU and 2 μM CpG-ODN groups, HepG2 cells had no obvious change. For 4 μM CpG-ODN group, cells began to shrink and the floating cells appeared in the culture medium. For 7.5 μg/ml 5-FU plus 2 μM or 4 μM CpG-ODN group, most of the HepG2 cells lost contacted with the surrounding cells and emerged more floating cells. Meanwhile, number of survival cells decreased significantly when compared with the medium group (Figure 2A). Nuclear stained with Hoechst 33258, revealed nuclear chromatin condensation in the CpG-ODN and 5-FU alone or together treated cells, which was typical of apoptotic cells. In 7.5 μg/ml 5-FU plus 2 μM or 4 μM CpG-ODN group, the change is more obviously, while the cells of medium group were diffusing uniform fluorescence (Figure 2B). The results of observation demonstrated that CpG-ODN in combination with 5-FU could affect the cell morphology of HepG2 cells and accelerate cell death.

### CpG-ODN in combination with 5-FU increases apoptosis in HepG2 cells

**Figure 3:**
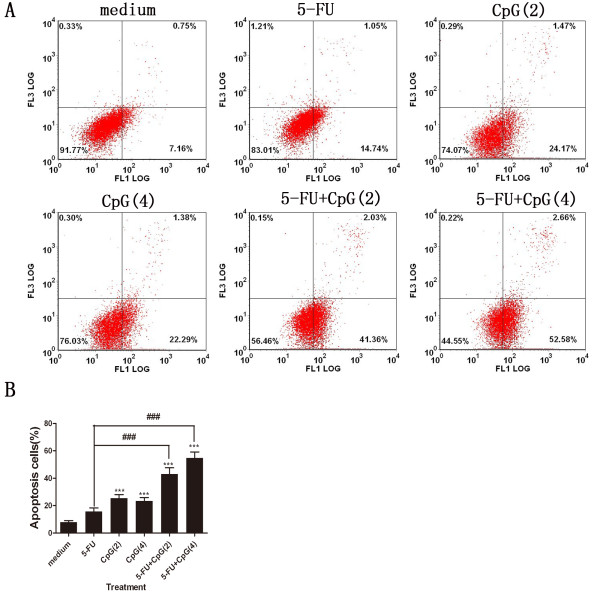
**Effect of CpG-ODN and/or 5-FU treatment on apoptosis of HepG2 cells. A**: The apoptosis was examined using annexin V-FITC/PI staining and flow cytometry analysis. One representative flow cytrometry analysis of apoptosis in HepG2 cells. Fluorescence intensity for annexin V/FITC is plotted on the x-axis, and PI is plotted on the y-axis. FITC^-^/PI^-^, FITC^+^/PI^-^, FITC^+^/PI^+^, FITC^-^/PI^+^ was regarded as living, early apoptotic, late apoptotic and necrotic cells, respectively. **B**: The percentage of apoptotic cells examined by annexin V-FITC/PI staining and flow cytometry analysis. Results are presented as mean ±SD of three separate experiments. ***P<0.001 versus medium. ^###^P<0.001 versus 5-FU. In this figure, 5-FU, CpG(2) and CpG(4) represent that the concentration of 5-FU, CpG-ODN and CpG-ODN is 7.5 μg/ml, 2 μM, 4 μM, respectively.

We investigated that the CpG-ODN and/or 5-FU treatment induced apoptosis using an Annexin V-FITC/PI staining method. To evaluate whether CpG-ODN promotes the chemosensitivity of 5-FU treated HepG2 cells by up-regulating apoptosis, the apoptotic rate of HepG2 cells following treatment with CpG-ODN and 5-FU alone or together for 48 h was detected. Fortunately, the result showed that CpG-ODN in combination with 5-FU treatment promoted apoptosis when compared with CpG-ODN and 5-FU alone treatment (Figure 3A and B). The finding suggested that CpG-ODN increased the chemosensitivity of 5-FU-treated HepG2 cells by inducing apoptosis.

### CpG-ODN in combination with 5-FU enhances cell cycle arrest at the S phase in HepG2 cells

**Figure 4:**
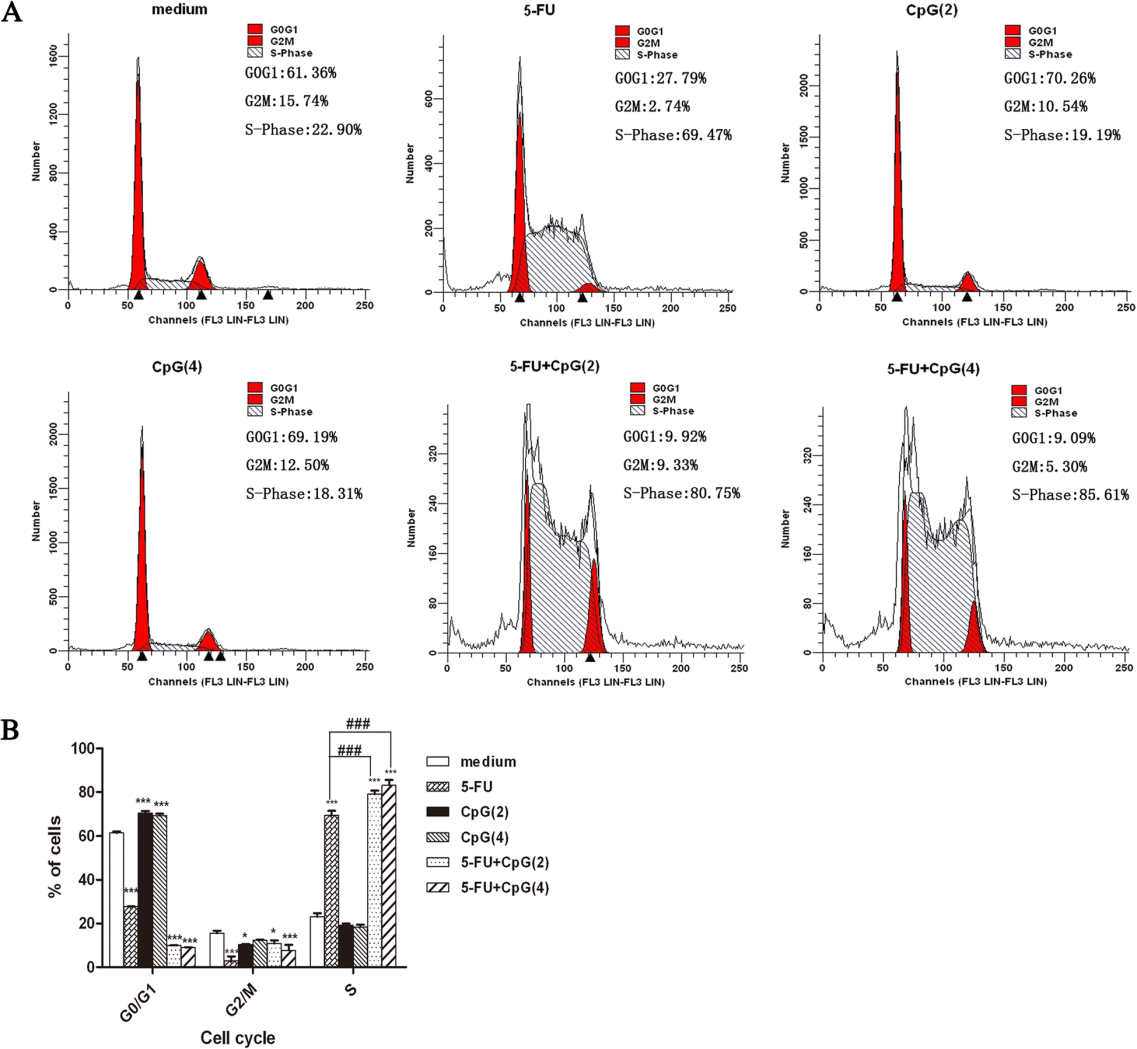
**Effect of CpG-ODN and/or 5-FU treatment on HepG2 cells cycle distribution. A**: Cells were incubated with CpG-ODN and 5-FU alone or together at indicated concentration for 48 h, and the cell cycle distribution was evaluated using PI staining and flow cytometry analysis. One representative flow cytometry analysis of cell cycle distribution. **B**: The percentage of cells in G0/G1, S and G2/M phase is expressed as mean ±SD of three independent experiments. *P<0.05,***P<0.001 versus medium. ^###^P<0.001 versus 5-FU. In this figure, 5-FU, CpG(2) and CpG(4) represent that the concentration of 5-FU, CpG-ODN and CpG-ODN is 7.5 μg/ml , 2 μM , 4 μM , respectively.

In order to examine whether the chemosensitivity of CpG-ODN was related to cell cycle arrest, we next measured the cell cycle in HepG2 cells with flow cytometry analysis and PI staining. Cells were incubated with CpG-ODN and 5-FU alone or together as indicated concentration for 48 h. As illustrated in Figure 4, the result showed that CpG-ODN alone slightly induced cells cycle arrest at G0/G1 phase and 5-FU alone could increase cells to enter S phase. Moreover, CpG-ODN in combination with 5-FU treatment caused drastic accumulation of cells in S-phase of the cell cycle, from 5-FU treatment (69.34±2.11) to CpG-ODN in combination with 5-FU treatment (83.20±2.40). The increase in S-phase cell population was accompanied by a concomitant reduction of cells in G0/G1 and G2/M phase of cell cycle. Therefore, CpG-ODN in combination with 5-FU could further induce cells cycle arrest at S phase when compared with 5-FU alone (Figure 4A and B). The result illustrated that the chemosensitizing effect of CpG-ODN was related to further induce cell cycle arrest at the S phase in 5-FU-treated HepG2 cells.

### CpG-ODN in combination with 5-FU promotes the chemosensitivity of 5-FU in HepG2 cells by down-regulating the mRNA expression of Livin and Survivin

**Figure 5:**
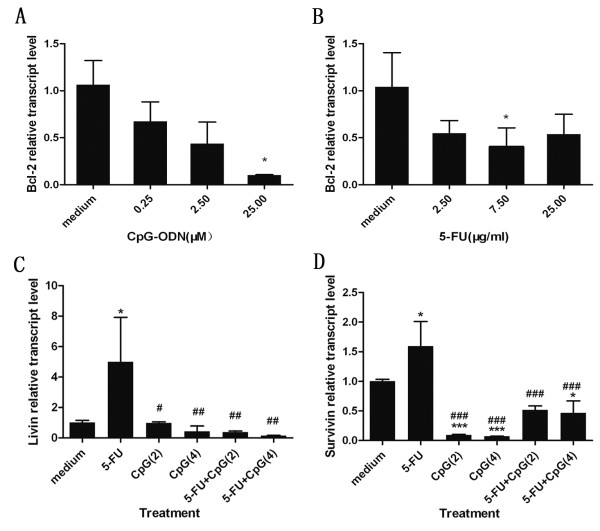
**Effect of CpG-ODN and/or 5-FU treatment on anti-apoptotic factors within HepG2 cells. A** and **B**: The cells seeded in six-well plates were treated with the indicated concentration of CpG-ODN (A) or 5-FU (B). After 48 h, total RNA was extracted for Bcl-2 mRNA expression using Real-time quantitative PCR. **C** and **D**: The cells seeded in six-well plates were treated with the indicated concentration of CpG-ODN and 5-FU alone or together, the Livin(C) and Survivin(D) mRNA expression were analyzed using Real-time quantitative PCR analysis as described above. Results are presented as mean ±SD of three separate experiments. *P<0.05, ***P<0.001 versus medium. ^#^P<0.05, ^##^P<0.01, ^###^P<0.001 versus 5-FU. In this figure, 5-FU, CpG(2) and CpG(4) represent that the concentration of 5-FU, CpG-ODN and CpG-ODN is 7.5 μg/ml, 2 μM, 4 μM, respectively.

The antiapoptotic factors of Bcl-2 family which were related to the tumorigenesis and the sensitivity of chemotherapeutic drugs in tumor. Overexpression of Bcl-2 protein is common in many human cancers, and contributes to resistance to chemotherapy [18]. In order to test whether CpG-ODN or 5-FU could affect the expression of Bcl-2 within HepG2 cells, cells seeded in six-well plates were treated with indicated concentration of CpG-ODN or 5-FU. After 48 h, total RNA was extracted for Bcl-2 mRNA expression using real-time PCR. The results showed that CpG-ODN and 5-FU alone could decrease the mRNA expression of Bcl-2 within HepG2 cells (Figure 5A and B). These results showed that CpG-ODN or 5-FU alone could induce the apoptosis of HepG2 cells by down-regulating the expression of Bcl-2.

The Livin and Survivin protein, belong to the inhibitors of apoptotic proteins (IAPs), were highly expressed in tumor tissue but lowly in normal tissue, and the induction of apoptosis was generally associated with downregulation of Survivin and Livin within tumor cells [19,20]. To examine whether CpG-ODN in combination with 5-FU promotes the chemosensitivty of 5-FU in HepG2 cells by regulating the expression of Livn and Survivin, The real-time PCR was tested with the same method as the above-mentioned. For the 5-FU (7.5 μg/ml) group, the relative mRNA expression level of Livin and Survivin is 4.98±2.94 and 1.59±0.42, respectively. The results showed that 5-FU alone could up-regulate the expression of Livin and Survivin. For the CpG-ODN(2 μM) group, the relative mRNA expression level of Livin and Survivin is 0.98±0.07 and 0.09±0.01, respectively. For the CpG-ODN(4 μM) group, the relative mRNA expression level of Livin and Survivin is 0.43±0.35 and 0.07±0.00, respectively. The results showed that CpG-ODN alone could down-regulate the expression of Livin and Survivin. For the 5-FU(7.5 g/ml)+CpG-ODN(2 μM) group, the relative mRNA expression level of Livin and Survivin is 0.38±0.07 and 0.52±0.07, respectively. For the 5-FU(7.5 g/ml)+CpG-ODN(4 μM) group, the relative mRNA expression level of Livin and Survivin is 0.14±0.02 and 0.47±0.20, respectively. The results showed that CpG-ODN in combination with 5-FU could down-regulate the expression of Livin and Survivin (Figure 5C and D). These clear results explained that CpG-ODN in combination with 5-FU could promote the chemosensitivity of 5-FU in HepG2 cells by down-regulating the expression of Livin and Survivin when compared with 5-FU treatment.

## Discussion

Convincing evidence from both animal experiments and pro-clinical studies suggest that CpG-ODN alone or in combination with other therapeutics are useful for the treatment of malignant tumors [21,22]. In the present experiment, we firstly demonstrated that CpG-ODN could significantly increase the chemosensitivity of 5-FU in HepG2 human hepatoma cells in vitro. The mechanism was related to CpG-ODN-mediated the expression of anti-apoptotic factors within tumor cells, then inducing apoptosis and cell cycle arrest at S phase. These findings provide new understanding of CpG-ODN-mediated direct cytotoxic effects and new insights into the application of chemosensitizer.

Currently, a number of clinical trials and animal experiments have confirmed that the activation of various immune cells and the production of cytokines induced by CpG-ODN have significant effect of anti-tumor [23,24]. CpG-ODN is considered as a potential chemosensitizer with unclear mechanism. Previous studies reported that Peritumoral CpG-ODN1826 treatment induces modulation of gene involved in DNA repair and sensitizes cancers cells to DNA-damaging cisplatin treatment in human IGROV-1 ovarian tumor cells [25], and peritumoral injection of CpG-ODN1826 in combination with subcutaneous injection of 5-FU inhibit tumor growth and reverse the immunosuppression caused by the therapy 5-fluorouracil in murine hepatoma [26]. However, the direct cytotoxicity toward HepG2 cells is not investigated in vitro. In our experiment, the results demonstrated that CpG-ODN could significantly increase the chemosensitivity of 5-FU in human hepatoma HepG2 cells in vitro.

Apoptosis was thought to be the major reason of cell death induced by chemosensitizer. Recent studies indicated that stimulation of TLR9 with CpG-ODN enhanced apoptosis in murine or human tumor cells [27,28], but ODN M362 promotes cell proliferation and survival in human hepatocellular carcinomas [10]. So we compare the direct cytotoxicity of CpG-ODN and ODN M362 toward HepG2 cells, our results showed that CpG-ODN induced significant inhibition of the survival of HepG2 cells, and ODN M362 had not direct cytotoxicity toward HepG2 cells (Figure 1). we next documented that apoptosis was responsible for CpG-ODN and/or 5-FU induced cytotoxicity of HepG2 cells using MTS assay, observation of cell morphology, Hoechst 33258 staining, and annexin V-FITC staining (Figures 1, 2 and 3), these results indicated that CpG-ODN increased the chemosensitivity of 5-FU in HepG2 cells by increasing apoptosis without the need for immune system of host. Although many studies were focused on the immunotherapeutic applications of CpG-ODN by modulating immune system of tumor-bearing hosts [29,30], some recent data showed that direct cytotoxicity against tumor cells is promising for therapy of different malignancies [31,32]. These previous studies strongly supported our study, these results showed that CpG-ODN directly induced apoptosis and increased the chemosensitivity of 5-FU in HepG2 human hepatoma cells.

Cell cycle arrest was thought to be another major reason of cell death induced by anti-tumor drugs [33,34]. Fluorouracil (5-FU) is a pyrimidine analogue which is transformed inside the cell into different cytotoxic metabolites and then incorporates into DNA and RNA, finally inducing cell cycle arrest and apoptosis by inhibiting the cell^’^s ability to synthesize DNA. It is an S-phase specific drug and only active during certain cell cycles. In this study, 7.5 μg/ml of 5-FU induced cell cycle arrest at S phase, which was in line with previous study [35]. Meanwhile, we found that 2 μM or 4 μM of CpG-ODN in combination with 7.5 μg/ml of 5-FU could further induce cells cycle arrest at S phase when compared with 5-FU alone (Figure 4). These findings suggest that CpG-ODN in combination with 5-FU induced apoptosis by interrupting the transition of cell cycle from S phase into G2/M phase, suggesting that the chemosensitizing effect of CpG-ODN was related to cell cycle arrest at S phase.

The upregulation of Bcl-2 expression caused resistance to chemotherapeutic drugs and radiotherapy, while the downregulation of Bcl-2 expression may promote apoptotic response to anticancer drugs [36,37]. Real-time quantitative PCR experiments showed that CpG-ODN and 5-FU alone inhibited the expression of Bcl-2 within HepG2 cells. Furthermore, CpG-ODN treatment suppressed the expression of Bcl-2 in a dose-dependent manner (Figure 5). These results suggested that the apoptosis induced by CpG-ODN and 5-FU is related to the downregulation of Bcl-2, but the exact molecular mechanism needs further study.

Previous studies have demonstrated that the overexpression of inhibitors of apoptotic proteins (IAPs) was resistant to the apoptosis induced by chemotherapy or radiotherapy [19,38,39], and Livin and Survivin protein belong to IAPs. Livin and Survivin produce anti-apoptotic effects through a complex signaling pathway [40,41]. Some studies have shown that overexpression of Survivin or Livin was closely related to chemoresistance, and inhibition of Survivin or Livin improved the sensitivity of tumor to chemotherapy [42-44]. In present study, our data showed that 5-FU alone could up-regulate the expression of Livin and Survivin within HepG2 cells (Figure 5). The result suggested that the expression of Livin and Survivin within HepG2 cells was induced by chemotherapy drug 5-FU, thus resisted to apoptosis induced by 5-FU. However, HepG2 cell following treatment with CpG-ODN (2 μM or 4 μM) in the presence or absence of 5-FU(7.5 μg/ml) could down-regulate the expression of Livin and Survivin (Figure 5) .These results suggested CpG-ODN could promote the chemosentivity of 5-FU in HepG2 cells by down-regulating the expression of Livin and Survivin within HepG2 cells. The said results provided a new field of view for the mechanism of chemosensitizing effect of CpG-ODN, which was not reported previously.

In conclusion, our results demonstrated that CpG-ODN possessed a chemosensitizing effect by down-regulating the expression of anti-apoptotic factors in HepG2 human hepatoma cells, leading to apoptosis and further inducing cell cycle arrest at S phase when compared with 5-FU treatment. Therefore, CpG-ODN may be a potential candidate as chemosensitizer for Hepatocellular carcinoma.

## Materials and methods

### Reagents

CellTiter 96®AQueous One Solution Cell Proliferation Assay was purchased from Promage (CA, USA). Propidium iodide (PI) and Rnase A were purchased from Sigma (NY, USA). Annexin V/FITC kit and Hoechst Staining Kit were purchased from Beyotime (Jiangsu, China). Trizol Reagent were purchased from Invitrogen(Carlsbad, CA, USA). M-MLV RTase cDNA Synthesis kit and SYBR® Premix Ex Taq™II kit were purchased from TaKaRa Biotechnology Co., Ltd. (Dalian, China). 5-Fluorouracil was purchased from Sigma (NY, USA).

All the primers were synthesized by Sangon Biotech Co., Ltd (Shanghai, China). CpG-ODN contains a total of 72 bases (5′-AA*CG*TTGT*CG*TCGA*CG*T*CG*T*CG*TCAGGCCTGA*CG*TTAT*CG* ATGG*CG*TTGT*CG*TCAA*CG*TTGT*CG*TTAA*CG*TT-3′). CpG-ODN, non-CpG-ODN (a convers- ed CG sequence of CpG-ODN) and ODN M362 (5′-T*CG*T*CG*T*CG*TT*CG*AA*CG*A*CG*TTGAT-3′) were synthesized by Sangon Biotech Co., Ltd (Shanghai, China). CG dinucleotides were indicated with underline. All oligodeoxynucleotides used in the experiments were in phosphorothioate backbone which was used to reduce susceptibility of the ODN to DNase digestion, thereby significantly prolonging its half-time in vitro. The oligodeoxynucleotides were dissolved with sterilized double distilled water.

### Cells culture

Human hepatoma HepG2 cells and BEL-7402 cells, human lung cancer A549 cells were cultured in 1640’s medium (Hangzhou Evergreen Biological Engineering Company, China) supplemented with 10% fetal bovine serum(FBS) and antibiotics (100 U/ml penicillin and 100 μg /ml streptomycin) in a 5% CO^2^ atmosphere at 37°C. Endotoxin levels in cell culture media and supernatants were undetectable(<1 ng/ml) as assessed by Limulus assay.

### Cell viability assay

Cells (2×10^3^/100 μl) were seeded in 96-well plates and treated on the following day with indicated concentration of CpG-ODN, 5-FU or CpG-ODN in combination with 5-FU. Cell viability was analyzed using CellTiter 96®AQueous One Solution Cell Proliferation Assay (Promage) according to the manufacture’s instructions, and optical density (OD) was read at 490 nm on a microplate reader (Bio-Rad, California, USA). The Viability(%) was calculated according to the following equation: Viability(%)=(OD _treated_/ OD _medium_)×100%. The inhibition rate was calculated according to the following equation: Inhibition rate(%)=(1- OD _treated_/ OD _control_)×100%.

### Cell morphology

Cells (1×10^5^/2 ml) were seeded in 6-well plates and grown for 24 h in order to attach to the surface of the plates completely. They were treated with indicated concentrations of CpG-ODN, 5-FU or CpG-ODN in combination with 5-FU. After incubation for another 48 h, Cell morphology was photographed by the inverted green light microscope (Olympus, Tokyo, Japan).

For another, after incubation, the medium were removed, the cells were rinsed PBS and stained using Hoechst Staining Kit according to the manufacture’s instructions. Stained nuclei were visualized under UV excitation and photographed using an Olympus fluorescence microscopy (Olympus, Tokyo, Japan).

### Apoptosis assay

Cells (1×10^5^/2 ml) were seeded in 6-well plates and treated on the following day with indicated concentrations of CpG-ODN, 5-FU or CpG-ODN in combination with 5-FU. After incubation for another 48 h, cells were trypsinized, washed with PBS, and stained using an Annexin V/FITC kit according to the manufacture’s instructions. Apoptosis was detected using a COULTER Epics xL Flow cytometer (Beckmam Coulter, CA, USA) within 1h after staining. Ten thousand events were evaluated for each sample. Data were analyzed using FCS Express Version 3 (De Novo Software, Canada).

### Cell-cycle phase distribution assay

Cells (1×10^5^/2 ml) were seeded in 6-well plates and treated on the following day with indicated concentrations of CpG-ODN, 5-FU or CpG-ODN in combination with 5-FU. After incubation for another 48 h, adherent and floating cells were collected and fixed in ice-cold ethanol (70% in ddH_2_O) at 4°C overnight. The cells were concentrated by removing ethanol and treated with 0.01% Dnase-free RNase A for 10 min at 37°C. Cellular DNA was stained with 0.02% propidium iodide (PI) for 30 min at 4°C in the dark. Cell cycle distribution was detected with FCM on a COULTER Epics xL flow cytometer (Beckmam Coulter, CA, USA). Ten thousand events were evaluated for each sample and the percentage of cells at specific phases was analyzed using ModFit LT software (Verity Software House, USA).

### Real Time PCR assay

Cells (1×10^5^/2 ml) were seeded in 6-well plates and treated on the following day with indicated concentration of CpG-ODN, 5-FU or CpG-ODN in combination with 5-FU. After incubation for another 48 h, total RNA was extracted using a Trizol reagent and then reversely transcribed into cDNA. The transcribed cDNA template was mixed with SYBR® Premix Ex Taq™ II kit and the following primers: Bcl-2, forward, 5′-CTTCGCCGAGATGTCCAGCCA-3^′^, reverse, 5′- CGCTCTCCACACACA TGACCC-3^′^; Livin, forward, 5′-TCTGAGGAGTTGCGTCTGG-3^′^, reverse, 5′-GCACCTCACCTTG TCCTGAT-3^′^; Survivin, forward, 5′-GACCACCGCATCTCTACATTC-3^′^, reverse, 5′-AAGTCT GGCTCGTTCTCAGTG-3^′^; β-actin, forward, 5′-TGACGTGGACATCCGCAAAG-3^′^, reverse, 5′-CTGGAAGGTGCACAGAGAGG-3^′^. Real-time PCR was performed using a Bio-Rad iQ5 quantitative PCR instrument (Bio-Rad, USA) with three-step Mothod as follows: Pre-denature at 95°C for 300s (1 cycle), denature at 95°C for 30s→anneal at 60°C for 20s →extend at 72°C for 45s(40 cycles), and an additional extension at 72°C for 7 min. Dissociation curve analysis was performed to see if there was any bimodal dissociation curve or abnormal amplification plot. For each sample, mRNA expression levels for specific transcripts were normalized to the amount of β-actin and 2^-△△Ct^ method was used to analyze the gene-expression data.

### Statistical analysis

Date was presented as mean ± standard deviation (SD). One-way ANOVA was used to test the differences between the treated and the control groups, followed by Tukey’s multiple comparisons. Differences with the p value less than 0.05 were considered as statistically significant.

## Competing interests

The authors declare that they have no competing interests.

## Authors' contributions

SL, GH and MM conceived and designed the experiments. SL participated in data collection, data analysis and drafted the manuscript. SL carried out flow cytometry analysis and Cell morphology assay. GH, LF and SH carried out Real Time quantitative PCR assay. JL and MZ carried out cell viability assay. All authors read and approved the final manuscript.
